# Effectiveness and safety of different doses of tenecteplase in the treatment of acute ischemic stroke: A protocol for systematic review and meta-analysis

**DOI:** 10.1097/MD.0000000000023805

**Published:** 2021-01-22

**Authors:** Guanfeng Chen, Chunfeng Bai, Zhou Zhu, Junheng Li, Sen Shao

**Affiliations:** XiXi Hospital of Hangzhou, Hangzhou, Zhejiang Province, China.

**Keywords:** acute ischemic stroke, intravenous thrombolysis, protocol, systematic review, tenecteplase

## Abstract

**Background:**

Tenecteplase is a modified recombinant tissue-plasminogen activator, which is effective and safe in the treatment of acute ischemic stroke. However, the therapeutic dose of tenecteplase has been controversial. The purpose of this study is to systematically investigate the efficacy and safety of different doses of tenecteplase thrombolytic therapy for acute ischemic stroke.

**Methods:**

Computer retrieval of English databases (PubMed, EMBASE, Web of Science, the Cochrane Library) and Chinese databases (CNKI, Wanfang, Viper, and Chinese Biomedical Database) is conducted for a randomized controlled clinical study on thrombolytic treatment of acute ischemic stroke with different doses of tenecteplase from the establishment of the database to October 2020. Two researchers independently conduct data extraction and literature quality evaluation on the quality of the included studies, and meta-analysis is conducted on the included literatures using RevMan5.3 software.

**Outcome:**

In this study, National Institute of Health Stroke Scale (NIHSS) score, Modified Rankin Scale (mRS) score scale, symptomatic intracranial hemorrhage (SICH) incidence, All-cause mortality, and so on are used to evaluate the efficacy and safety of tenecteplase thrombolytic therapy in acute ischemic stroke with different doses.

**Conclusion:**

This study will provide reliable evidence-based evidence for the clinical application of different doses of tenecteplase in thrombolytic therapy for acute ischemic stroke.

**OSF Registration number:**

DOI 10.17605/OSF.IO/2MPCW.

## 1 Introduction

Stroke is the second leading cause of death after malignant tumors. Every year, about 22 million people around the world suffer from stroke, mainly including 3 categories: ischemic (87%), hemorrhagic (10%), and subarachnoid hemorrhage (3%).^[1]^ Sixty percent of acute ischemic stroke (AIS) is caused by thrombosis, and 40% is caused by embolism. The lifetime risk of stroke for patients aged 55 to 75 years is 20% in women and 15% in men. Moreover, about 10% of patients with AIS die within 1 year, and 20% to 25% of patients suffer from severe disability.^[2]^

The key point of treatment of AIS is to achieve blood vessel recanalization and restore brain tissue irrigation effectively in early stage,^[3]^ and timely intravenous thrombolysis could make blocked blood vessels recanalize, thereby saving ischemic pentheus and finally reducing the mortality and disability rate of ischemic stroke.^[4]^ Recombinant tissue-plasminogen activator (RT-PA) and intravenous thrombolysis within a time window are currently internationally recognized as effective treatment methods for acute ischemic stroke.^[5]^ Alteplase, the active ingredient in the recombinant tissue-type plasminogen activator (RT-PA), can activate the conversion of plasminogen into plasmin to achieve a good thrombolytic effect, while tenecteplase is a biologically engineered variant of alteplase, which has a longer t1/2 and stronger anti-plasminogen activator inhibitor effect. In addition, tenecteplase is cheaper and convenient to administer.^[6]^ At present, the clinical efficacy of intravenous thrombolysis with different doses of tenecteplase has not been clearly defined. A non-randomized dose-increasing safety study has shown that tenecteplase of 0.1 to 0.4 mg/kg is safe for ischemic stroke,^[7]^ but there is no systematic evaluation of which dose is more beneficial for the treatment of acute ischemic stroke.

At present, there have been a number of randomized controlled trials^[8–10]^ on the doses of tenecteplase, ranging from 0.1 to 0.4 mg/kg, but the results are uneven. Therefore, this plans to systematically evaluate the effectiveness and safety of intravenous thrombolysis of AIS with different doses of tenecteplase, so as to provide a reliable reference basis for clinical application.

## 2 Methods

### 2.1 Protocol register

This study of systematic review and meta-analysis has been drafted under the guidance of the preferred reporting items for systematic reviews and meta-analyses protocols (PRISMA-P).^[11]^ It has been registered on open science framework (OSF) on November 14, 2020. (Registration number: DOI 10.17605/OSF.IO/2MPCW).

### 2.2 Ethics

Since this is a protocol with no patient recruitment and personal information collection, the approval of the ethics committee is not required.

### 2.3 Eligibility criteria

#### 2.3.1 Types of studies

We will collect a randomized controlled study of tenecteplase at different doses in the treatment of AIS in Chinese and English only.

#### 2.3.2 Research objects

Patients who are clinically diagnosed as AIS and confirmed by brain computed tomography (CT) and magnetic resonance imaging (MRI), with no limitation on nationality, race, age, sex, and course of disease.

#### 2.3.3 Intervention measures

The observation group receive intravenous thrombolysis with different doses of tenecteplase (including 0.10, 0.25, 0.40 mg/kg). The control group receive intravenous thrombolytic therapy with alteplase 0.9 mg/kg.

#### 2.3.4 Outcome indicators

(1) Primary outcome: National Institute of Health Stroke Scale (NIHSS) (after treatment, if the NIHSS score decreased by ≥8 points or 0–1 points after 24–48 hours, it would be considered as the improvement of early neurological function). Modified Rankin Scale (mRS) rating scale (90 days since the onset of disease, in which mRS 0–1 indicates a good prognosis and mRS 0–2 indicates the ability to live independently);
(2) Secondary outcomes: symptomatic intracranial hemorrhage (SICH) risk, (incidence of SICH as defined by the European Cooperative Acute Stroke Study (ECASS)II standard^[12]^). All-cause mortality.

### 2.4 Exclusion criteria

(1) Studies with incomplete data and repeated publication;
(2) Articles which are published as abstracts and whose author are unable to offer data after being contacted;
(3) Studies with no relevant outcome indicators or obvious error in the research data;
(4) The risk assessment of bias is the literature with high risk of bias.

### 2.5 Retrieval strategy

The computer searches CNKI, WanFang, Viper, Chinese Biomedical Literature Database, and Chinese search terms are “Ti Nai Pu Mei (TNK-tPA)” or “Zhong Feng (Stroke)” or “Ji Xing Que Xue Xing Nao Zu Zhong (Acute ischemic stroke)” or “Ji Xing Nao Geng Si (Acute cerebral infarction),” and “Ji Xing Nao Geng Se (Acute cerebral infarction).” Retrieval in English databases including PubMed, EMBASE, Web of Science, the Cochrane Library, Search terms in English are tenecteplase, TNK-tPA, acute ischemic stroke, AIS. The retrieval time is from the establishment of the database to October 2020, and all domestic and foreign literatures on different doses of tenaiprase for AIS treatment are collected. Take PubMed as an example, and the retrieval strategy is shown in Table 1.

**Table 1:** Search strategy in PubMed database.

Number	Search terms
#1	tenecteplase [Title/Abstract]
#2	TNK-tPA [Title/Abstract]
#3	#1 OR #2
#4	Stroke [MeSH]
#5	Cerebrovascular Stroke [Title/Abstract]
#6	Stroke, Cerebrovascular [Title/Abstract]
#7	Brain Vascular Accident [Title/Abstract]
#8	Acute Cerebrovascular Accident [Title/Abstract]
#9	acute ischemic stroke [Title/Abstract]
#10	Cerebrovascular Accidents, Acute [Title/Abstract]
#11	AIS [Title/Abstract]
#12	#4 OR #5 OR #6 OR #7 OR #8 OR #9 OR #10 OR #11
#13	#3 AND #12

### 2.6 Data filtering and extraction

**Figure 1:**
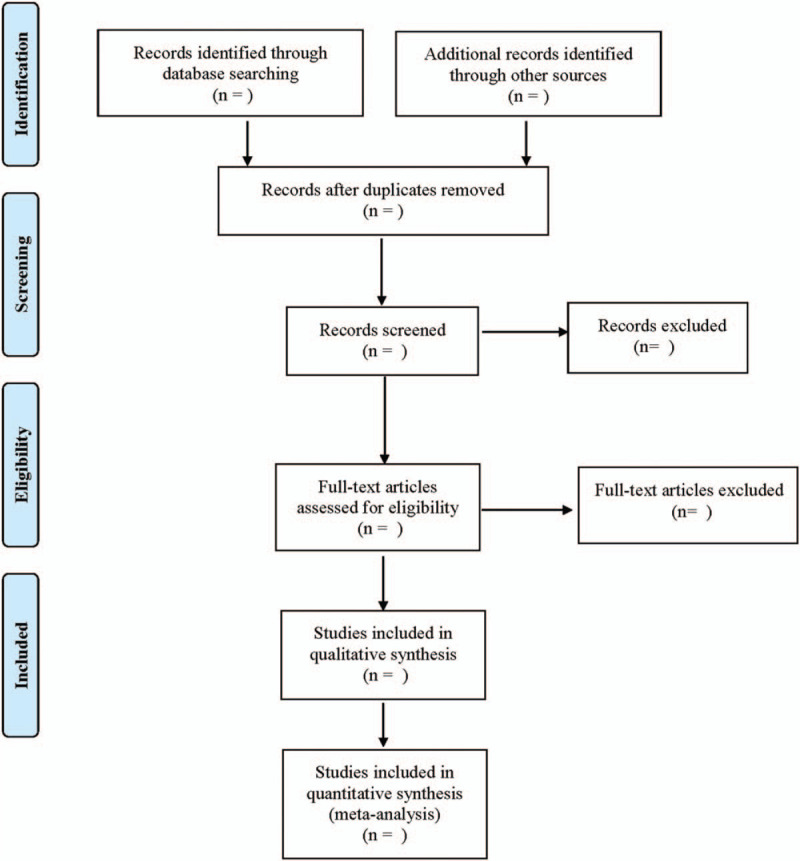
Flow diagram.

Data are extracted independently by 2 researchers and cross-checked. If there are different opinions, they would be discussed or solved with the assistance of a third researcher. The extracted contents include the first author, year of publication, country, study start and end time, number of cases, intervention measures, treatment time window, and outcome indicators. The literature selection process is shown in Fig. 1.

### 2.7 Literature quality evaluation

The Cochrane Risk Bias Assessment Tool is used to assess the risk bias of the included studies.^[13]^ According to the performance of the included literature in the above evaluation items, the 2 researchers will give low risk, unclear and high risk judgments one by one, and cross-check after completion respectively. In case of any disagreement, discussion will be carried out. If no agreement can be reached, discussion will be made by the researchers in the third party.

### 2.8 Statistical analysis

#### 2.8.1 Data analysis and processing

RevMan5.2 software is used for Meta-analysis. Odds ratio (RR) is used as the combined statistic for counting data, and standardized mean difference (SMD) is used as the combined statistic for measurement data. Point estimate and 95% confidence interval (95% CI) are given for each effect. Heterogeneity among study results is analyzed by chi-squared test. When *P* ≥ .10 and *I*^2^ ≤ 50%, heterogeneity is low. Fixed-effect model is used for meta-analysis. When *P* < .1, *I*^2^ > 50% indicates inter-study heterogeneity, and the source of heterogeneity should be analyzed. Clinical heterogeneity is treated by subgroup analysis. If no significant clinical or methodological heterogeneity can be found, statistical heterogeneity would be considered, and the random-effect model would be used for analysis. If the clinical heterogeneity is too obvious and the subgroup analysis cannot be performed, the meta-analysis would not be performed, but descriptive analysis is only performed.

#### 2.8.2 Dealing with missing data

If there is missing data in the article, contact the author via email for additional information. If the author cannot be contacted, or the author has lost relevant data, descriptive analysis will be conducted instead of meta-analysis.

#### 2.8.3 Subgroup analysis

Subgroup analysis is carried out according to the dose and treatment of tenecteplase.

#### 2.8.4 Sensitivity analysis

In order to determine the stability of outcome indicators, sensitivity analysis is used to analyze each outcome indicator.

#### 2.8.5 Assessment of reporting biases

Funnel plots will be used to assess publication bias if no fewer than 10 studies are included in an outcome measure. In addition, Egger and Begg test were used for the evaluation of potential publication bias.

#### 2.8.6 Grading the quality of evidence

We grade the outcome indicators through the Grading of Recommendation Assessment, Development and Evaluation (GRADE).^[14]^ The evaluation includes bias risk, interconnectedness, inconsistency, inaccuracy, and publication bias. The quality of evidence will be rated as high, medium, low, or very low.

## 3 Discussion

With the aging of the population, acute ischemic stroke has become one of the main diseases leading to death and disability, and the trend is increasing year by year, seriously affecting the physical health and quality of life of residents.^[15,16]^ There are many pathological mechanisms of ischemic stroke, among which acute arterial thrombosis leads to vascular occlusion is a common cause, and venous thrombolysis is the most important measure to restore blood flow.^[4]^

The emergence of tenecteplase as the third generation of thrombolytic drugs enriches and improves the clinical selection of thrombolytic drugs, with the following advantages^[17–19]^: Convenience: compared with the traditional alteplase (RT-PA) usage (0.9 mg/kg, 90 mg at most, 10% of the total amount is stably pushed within 1 minute, and the remaining 90% is stably dropped for 1 hour), tenecteplase has a longer half-life and only needs a single injection within 10 seconds; Efficacy: Compared with RT-PA, it has higher specificity to fibrin and higher recanalization rate; compared with common RT-PA, tenecteplase has higher specificity for fibrin at thrombus site, and its activity can be inhibited by endogenous inhibitors; compared with alteplase, it is cheaper to reduce the financial concerns of patients before use, thus increasing the proportion of patients with thrombolysis. Although it has been proved that tenecteplase is as effective as alteplase,^[20]^ the issue of dosage has been controversial.^[21]^ Studies have shown that 0.40 mg/kg tenecteplase is no more advantageous than 0.25 mg/kg tenecteplase in patients with large vessel occlusie ischemic stroke,^[22]^ indicating that dose size is not positively correlated with benefit, so it is necessary and urgent to explore the most appropriate dose.

According to the trial of the National Institute of Neurological Disorders and Stroke (NINDS) in the United States, the recommended dose of IV-TPA is 0.9 mg/kg (maximum 90 mg).^[23]^ The efficacy has been verified clinically after extensive clinical use,^[24]^ so it is reliable to evaluate the efficacy and safety of tenecteplase in different doses as a control. This systematic evaluation will evaluate the reliability of the efficacy of tenecteplase in the treatment of acute ischemic stroke at different doses based on available evidence, providing an evidence-based basis for the selection of clinicians.

However, this systematic review has some limitations. At present, there are few randomized controlled studies on tenecteplase doses, which may lead to publication bias due to the small number of included studies. At the same time, due to the limitation of language ability, we only search literature in English and Chinese, and may ignore research or reports in other languages.

## Author contributions

**Data curation:** Guanfeng Chen, Chunfeng Bai.

**Funding acquisition:** Sen Shao.

**Literature retrieval:** Guanfeng Chen and Chunfeng Bai.

**Software:** Zhou Zhu, Junheng Li.

**Supervision:** Sen Shao.

**Writing – original draft:** Guanfeng Chen, Chunfeng Bai.

**Writing – review & editing:** Guanfeng Chen, Sen Shao.
